# Coincidence of Persistent Müllerian duct syndrome and testicular tumors in dogs

**DOI:** 10.1186/s12917-017-1068-6

**Published:** 2017-06-02

**Authors:** Eun Jung Park, Seok-Hee Lee, Young-Kwang Jo, Sang-Eun Hahn, Do-Min Go, Su-Hyung Lee, Byeong-Chun Lee, Goo Jang

**Affiliations:** 1 Laboratory of Theriogenology & Biotechnology, College of Veterinary Medicine and the Research Institute of Veterinary Science, Seoul National University, Kwanak-ro 1, Daehak-Dong, Kwanak-Gu, Seoul, 08826 Republic of Korea; 2 Veterinary Teaching Hospital, College of Veterinary Medicine and the Research Institute of Veterinary Science, Seoul National University, Seoul, 08826 Republic of Korea; 3 Veterinary Pathology, College of Veterinary Medicine and the Research Institute of Veterinary Science, Seoul National University, Seoul, 08826 Republic of Korea; 4 Emergence Center for Food-Medicine Personalized Therapy System, Advanced Institutes of Convergence Technology, Seoul National University, Gyeonggi-do, 443-270 Republic of Korea

**Keywords:** Cryptorchidism, Hyperestrogenemia, PMDS, Prostatomegaly, Testicular tumor

## Abstract

**Background:**

Persistent Müllerian duct syndrome (PMDS), a rare form of male pseudohermaphroditism in dogs, is an abnormal sexual phenotype in males that is characterized by the existence of a hypoplastic oviduct, uterus, and cranial part of the vagina. Dogs suffering from PMDS are often accompanied by cryptorchidism. To date, it has been mainly found in the Miniature Schnauzer breed.

**Case presentation:**

In this report, two cases of PMDS with a malignant testicular tumor originating from cryptorchidism in breeds other than the Miniature Schnauzer breed are described. The patients were a seven-year-old male Maltese dog and a 17-year-old male mixed-breed dog weighing 3.8 kg. They also exhibited an enlarged prostate with or without abscess and an elevated serum estradiol level and were surgically treated to remove the testicular tumor and Müllerian duct derivatives.

**Conclusions:**

It is recommended that PMDS should be differentially diagnosed by ultrasonography and that orchiectomy be performed at an early age in patients suspected to have cryptorchidism to prevent the ectopic testes from becoming tumorous.

## Background

Persistent Müllerian duct syndrome (PMDS), a type of male pseudohermaphroditism, is a known autosomal recessive inherited abnormality in the Miniature Schnauzer. This syndrome refers to male dogs presenting normal karyotypes (78, XY) that have bilateral testes with vestigial organs of the oviduct, uterus, and cranial vagina, as the Müllerian duct has failed to regress [1, 2]. The regression of the Müllerian duct is controlled by Müllerian-inhibiting factor (MIF). Sertoli cells in the testes secrete MIF in normal and PMDS-affected fetuses and neonatal dogs [3], and a single base-pair mutation in the gene coding the canine MIF receptor impairs the regression of the Müllerian duct [4]. These observations indicate that PMDS results from the refractory targeting of cells by MIF rather than from a lack of MIF secretion.

Cryptorchidism often occurs simultaneously with PMDS, and the incidence rate of cryptorchidism is approximately 50% in PMDS dogs [2, 5]. Testicular tumors occur in both scrotal and ectopic testes, but ectopic testes tend to develop tumors more frequently than scrotal testes. PMDS with simultaneously existing testicular tumor in a Miniature Schnauzer dog has been reported [6], but there have been no reports for other breeds to our knowledge. Here, we report two cases of PMDS accompanied by testicular tumor from abdominal cryptorchidism in non-Miniature Schnauzer breeds. Informed consent was obtained from the owners.

## Case presentation

### Case 1

**Fig. 1:**
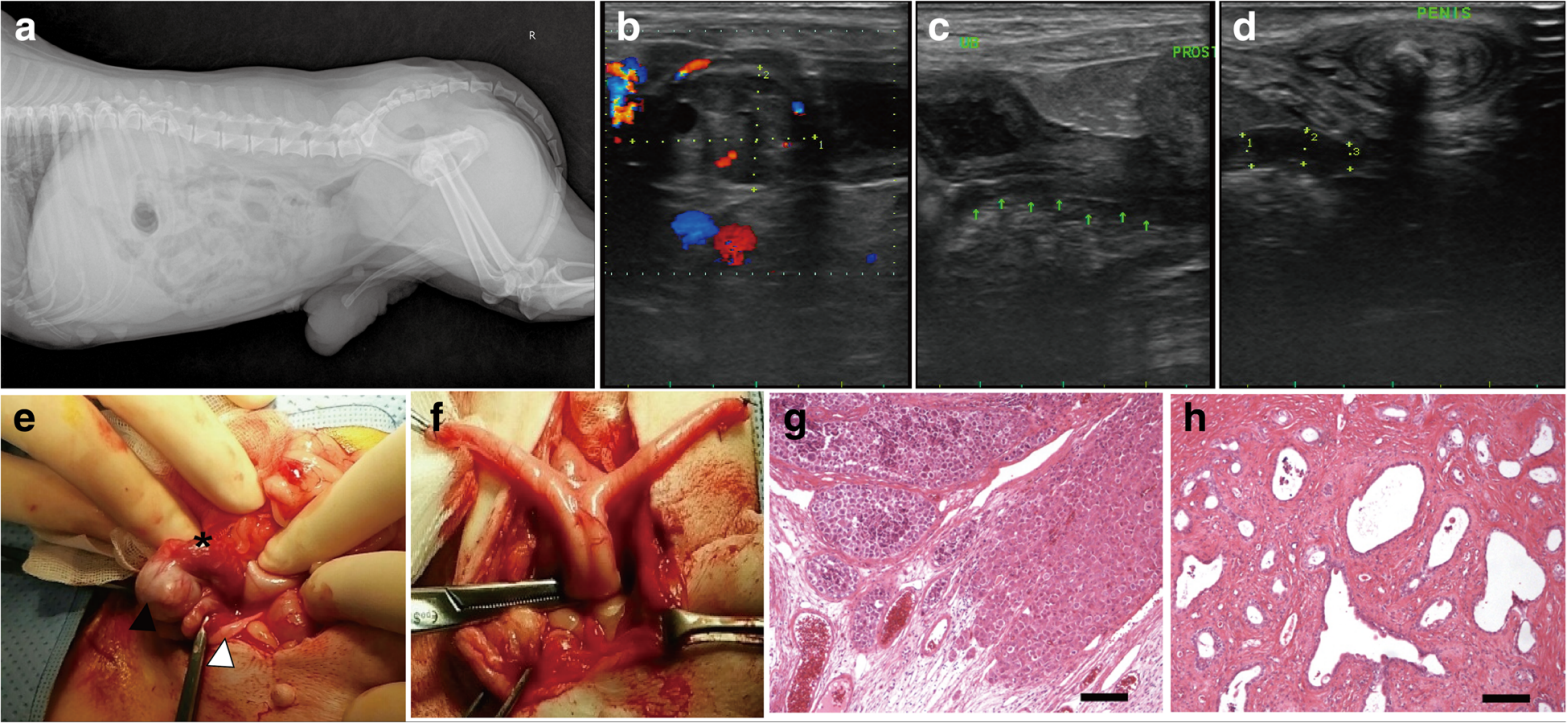
Radiography and ultrasonography findings in surgery and histological examination of the testicular tumor and Müllerian duct derivatives of Case 1. **a** prostatomegaly shown in radiography; **b** abdominal mass showing mild blood signal; **c** and **d** tubular structure with hypoechoic lumen (*arrows*) extending from the mass to adjacent inguinal region; **e** the abdominal mass (*filled arrowhead*), spermatic cord-like structure (pampiniform plexus and ductus deference, *asterisk*), and tubular structure (*blank arrowhead*); **f** smooth muscle structure with Y shape linked to the mass; **g** malignant seminoma with tumor emboli (× 100); **h** Müllerian duct derivatives involving thick muscle and lumen lining with epithelium (× 100). Scale bar = 200 um

A seven-year-old male Maltese dog that had been diagnosed with an abdominal mass was referred to the Veterinary Medical Teaching Hospital, Seoul National University, for medical treatment and surgical removal of the mass. An edematous, pendulous prepuce and enlarged nipples were observed during a physical examination. The owner reported that the dog had been castrated at 10 months old, and the veterinarian and owner agreed that both testes had been removed. In radiography and ultrasonography, prostatomegaly (1.50 × 0.94 cm) (Fig. 1a) and a round-shaped mass (2.14 × 1.36 cm) at the right cranial to the urinary bladder apex region were detected (Fig. 1b), respectively. A hypoechoic tubular structure with a diameter of 0.38 cm was found incidentally, extending from the mass to the right inguinal region (Fig. 1c and d). All the blood analysis panel values were within the normal reference intervals. Table 1 shows that the serum estradiol concentration was 19.6 pg/mL, which is slightly higher than the upper margin of the reference intervals in male dogs (<15 pg/mL [6, 7]), and the testosterone level was 0.025 ng/mL, which is much lower than in intact males (1–5 ng/mL) and similar to that of castrated dogs (<0.02 ng/mL [7]). Thus, we concluded that the mass in the abdomen secreted estradiol, not testosterone. For removing the mass, a laparotomy was performed. As the mass was exposed, it was found that a pampiniform plexus-like vascular structure and a ductus deferens-suspected tubular structure were connected to the mass (Fig. 1e). On the opposite site of the adnexa in the mass, a uterus-like smooth muscle structure with a Y shape linked to the mass was also found (Fig. 1e and f). The Y-shaped structure had vascular distribution and a rugged broad ligament, while the end of the left horn of the Y shape was suspended from the abdominal wall by fibrous tissue, and an incompletely fused body terminated at the prostate. The mass with adnexa was isolated from the abdomen and examined by a histologist. The sections confirmed the presence of malignant seminoma with tumor emboli in the testis (Fig. 1g) and thick muscle and lumen lining with epithelium, which were diagnosed as Müllerian duct derivatives (Fig. 1h). Four days after the surgery, the estradiol level decreased to less than 5 pg/mL.

**Table 1:** Blood analysis and serum sex hormone levels of both cases

		Case 1	Case 2	Reference range
CBC	WBC (/μL)	9490	5840	6000–17000
	Hct (%)	41.9	24.1	37.0–55.0
	Thrombocyte (/μL)	337,000	39,000	200,000–500,000
Na+/K+/Cl- (mEq/L)		149/4.5/116	155/3.9/118	145–155/2.7–5.0/96–122
PT/APTT (s)		-	12/ 89	11–17/ 72–102
Estrogen (pg/mL)		19.6	267.8	<15 [6, 7]
Testosterone (ng/mL)		0.025	0.602	1–5 [7]

### Case 2

**Fig. 2:**
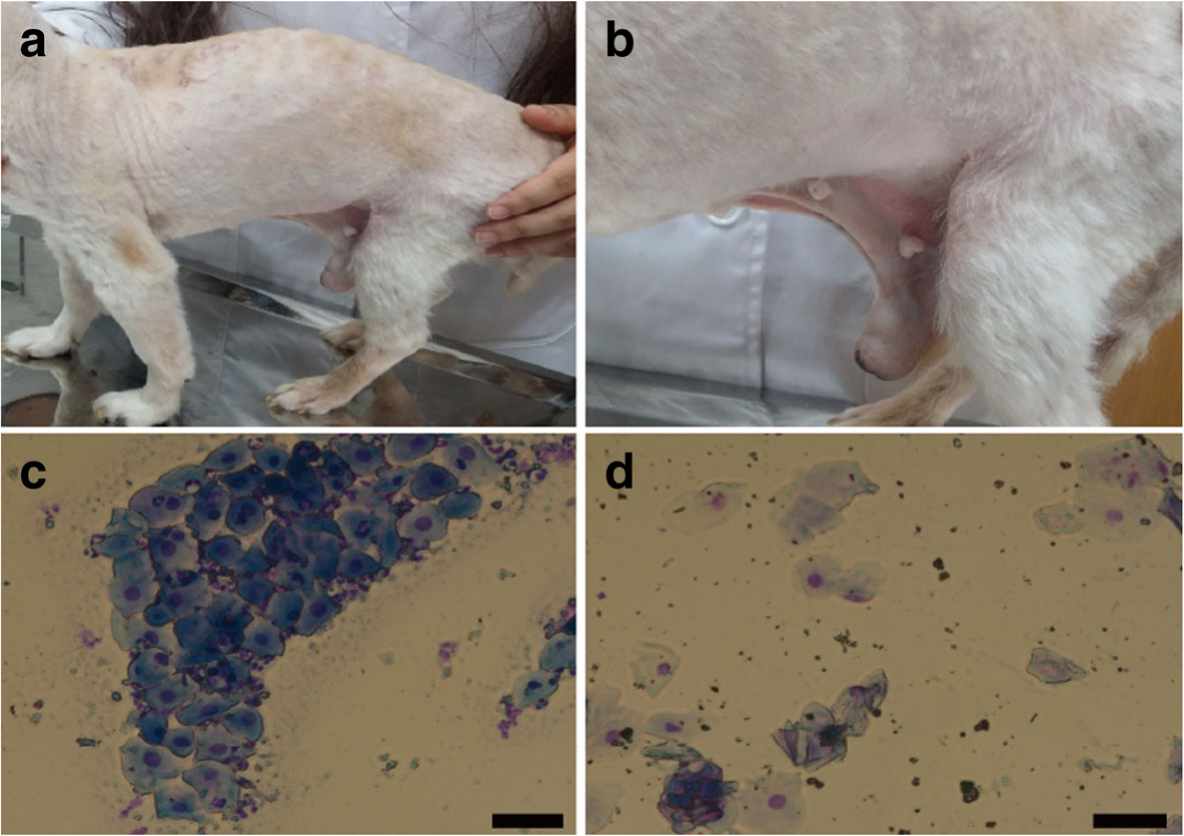
Photographs from physical examination and preputial cytology of Case 2. **a** hyperkeratosis and alopecia in trunk and thoracolumbar kyphosis; **b** pendulous prepuce and enlarged nipples; **c** nucleated round epithelial cells and neutrophils in preputial cytology of normal male dog (× 200); **d** superficial epithelial cells in preputial cytology of Case 2 (× 200). Diff-quik stain was used for the cytology. Scale bar= 50 um

**Fig. 3:**
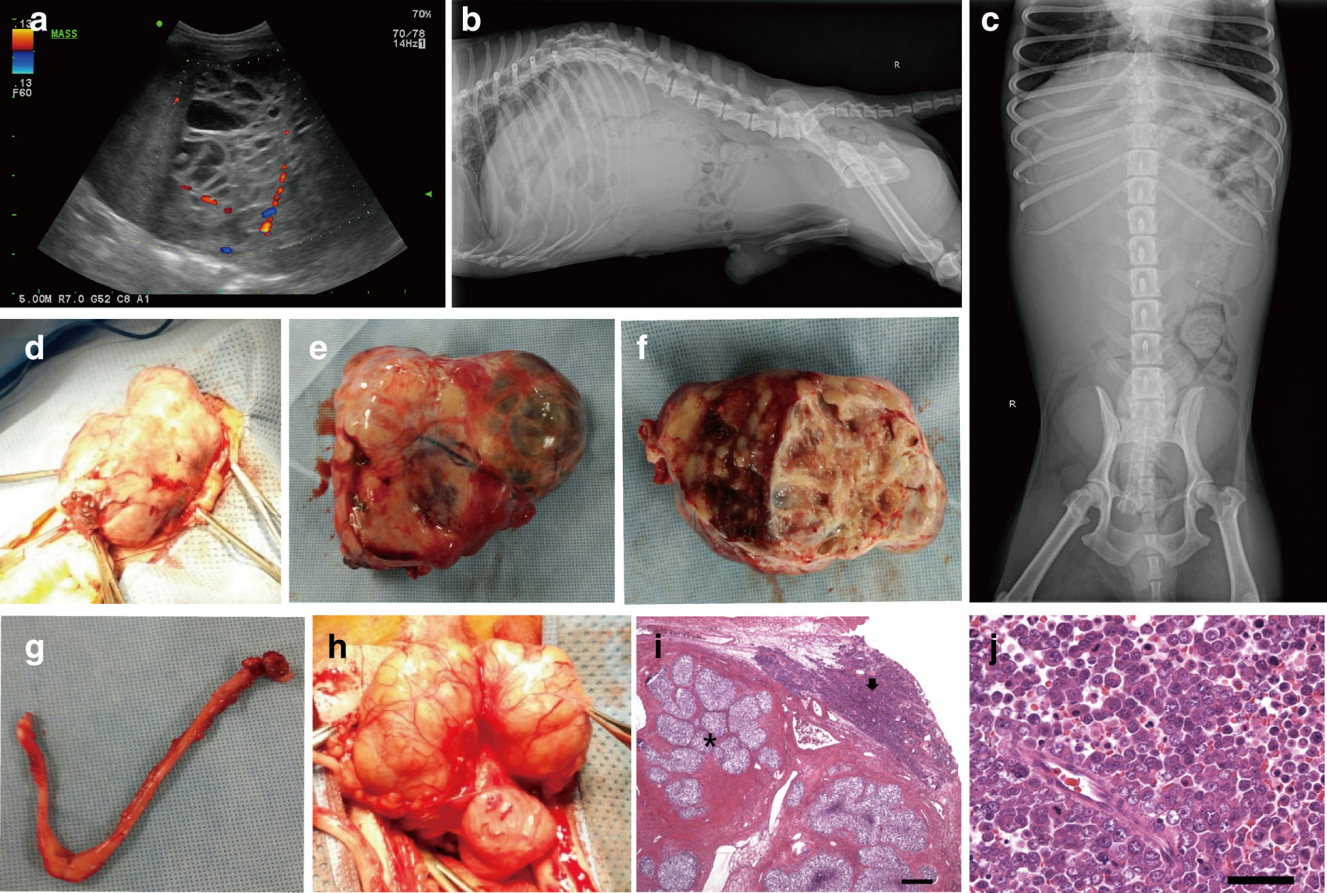
Ultrasonography and abdominal radiography findings in surgery and histological examination of the testicular tumor of Case 2. **a** abdominal mass including multiple cystic lesions with various echogenicities and blood signals. **b** and **c** kyphosis and ill-defined margin, marked homogenous soft tissue opacity of a mass in the right-middle abdomen (11 × 6 cm); **d**, **e**, and **f** exposed, removed, and dissected abdominal mass, respectively; **g** smooth muscle structure with Y shape connected to the mass; **h** urinary bladder that failed to distend and prostate containing pus; **i** collision tumor including malignant seminoma (*arrow*) and Sertoli cell tumor (*asterisk*) (× 12.5), scale bar = 400 um; **j** malignant seminoma (× 400), scale bar = 5 um

A 17-year-old male mixed-breed dog weighing 3.8 kg was transferred for diagnosis and medical treatment related to a very large abdominal mass. Hyperkeratosis, alopecia in the trunk and ear margin, a pendulous prepuce, enlarged nipples, unilateral scrotal testis, thoracolumbar kyphosis (Fig. 2a and b), and frequent urination were observed during a physical examination. A preputial swab showed an exfoliative cytology (Fig. 2d) similar to the vaginal cytology of female dogs in estrus, which is different from that of normal males (Fig. 2c). An ill-defined margin and marked homogenous soft-tissue opacity of a mass (11 × 6 cm) in the right-middle abdomen resulted in left cranial displacement of a descending, transverse colon and stomach and left caudal displacement of the intestinal loops (Fig. 3b and c). The additional relatively well-defined homogenous soft-tissue opacity of a mass (6.4 × 4.3 cm) in the caudal abdomen at the L5 pelvic inlet level causing cranial displacement of the intestinal loops was detected via radiography (Fig. 3b and c). Ultrasonography showed two severely enlarged, heterogenous masses in the abdomen. The larger abdominal mass included multiple cystic lesions in the parenchyma with echogenic and anechoic fluid and moderate blood signals (Fig. 3a). Information about the other abdominal organs taken from the ultrasonography was limited due to the volume of the masses. The blood analysis results presented pancytopenia (Table 1). The serum estradiol concentration was 267.8 pg/mL, which is approximately 18 times higher than the upper margin of the reference interval. The testosterone level was 0.602 ng/mL, which is lower than the normal reference interval in intact males (Table 1) and within the reference range of a dog with a Sertoli cell tumor (SCT) (0.1–2 ng/mL [7]). Therefore, the mass in the abdomen was suspected to be an SCT secreting estradiol strongly. Despite the risk of complications during and after surgery due to pancytopenia and the old age of the patient, the owner wanted the mass to be removed to relieve the discomfort and complications caused by the mass. Thus, the coagulation time, such as PT/APTT, was analyzed for a laparotomy, and these values were in the normal range (Table 1). The mass was too large to be extracted via a routine laparotomy; accordingly, the incision line was extended to the xiphoid process cranially. The diaphragm was abnormally pushed forward by the mass which occupied almost the entire abdominal cavity. Stay sutures were applied to the mass to remove it from the abdominal cavity carefully (Fig. 3d). The mass was removed successfully, and then dissected (Fig. 3e and f). A pampiniform plexus-like vascular structure and a smooth muscle structure with a Y shape linked to the mass were found (Fig. 3g), and one of its horns was isolated from the mass. While the other horn remained intact, we moved to the caudal abdomen and found the urinary bladder was collapsed and unable to distend as well as bilateral prostatic enlargement with abscess (Fig. 3h). The dog became hypotensive during the removal of the prostatic abscess, so the surgical procedure, including removal of the entire uterus-suspected structure and draining of pus in the prostate, was stopped and transfusion, dopamine, dobutamine, and vasopressin were administered to overcome the hypotension. The patient recovered from hypotension, but cardiopulmonary arrest suddenly occurred 36 h after surgery. Although the cardiac and pulmonary function was recovered by cardiopulmonary cerebral resuscitation, the patient failed to recover his brain function and died within 12 h. Histologic examination of the suspected testicular tumor mass allowed diagnosis of the mass as a collision tumor of malignant seminoma with SCT (Fig. 3i and j).

## Discussion

In this case report, two dogs presenting with PMDS and testicular tumor from cryptorchidism have been described. Both cases in this report exhibited elevated serum estradiol levels, testicular tumors, and feminization characteristics, including gynecomastia and a pendulous penile sheath. PMDS-affected dogs exhibit cryptorchidism frequently and may present with a fluid-filled uterus, urinary tract infection, or prostatic infection [8]. Less than 10% of seminomas produce estrogen (communicated with reviewer’s comment), so it is interesting that the malignant seminoma in Case 1 produced slightly more estradiol than in normal males. However, we had the limitation of having no standard because the GnRH stimulation test and multiple-sample assays of estradiol and testosterone were not conducted in this study.

The intra-abdominal testes have a significantly higher risk of being tumorous, especially seminoma and SCT, which have an approximately nine-fold incidence rate compared with scrotal testes [9]. Among the testicular tumor types, SCT specifically secretes excess estrogen, resulting in the feminization of male dogs and bone-marrow suppression, which leads to pancytopenia. Both patients presented elevated serum estradiol levels, but the patient in Case 2 only exhibited pancytopenia following severe hyperestrogenemia. Although the complications of pancytopenia include infection, anemia, and spontaneous bleeding, there was no spontaneous bleeding or delayed coagulation in this case, which enabled us to decide to remove the mass to relieve the patient from distress. As we confirmed, however, a severe prostatic abscess existed. It is assumed that prolonged pancytopenia resulting from hyperestrogenemia caused prostatic infection, which subsequently developed into an abscess. Regarding another effect of hyperestrogenemia, the preputial cytology of Case 2 exhibited exfoliative, superficial epithelial cells similar to the vaginal cytology of female dogs in estrus, which corresponds with a report that the preputial cytology of male dogs that have hyperestrogenemia (>40 pmol/L) exhibited superficial epithelial cells [10]. Recently, it has been reported that neoplastic Sertoli cells in dogs produce a significantly high MIF level compared with healthy Sertoli cells [11, 12]. If we had detected the level of AMH in both cases, the patient in Case 2, which had SCT, might have exhibited a higher level of AMH compared with the patient in Case 1 with no SCT. MIF secretion in canine immature Sertoli cells starts from embryonic day 34, when testicular differentiation initiates, and continues until day 143 after birth. Müllerian duct regression begins shortly after testicular development at day 36–46 of gestation through MIF in canines [13]. The PMDS in both cases of this report might have arisen from the abnormal action of MIF during the embryonic Müllerian duct regression period. Meyers-Wallen et al. report that oviduct regression depends on MIF activity, but uterus regression is independent of the MIF level [13]. That is, uterine and oviductal sensitivities to MIF differ, which explains why we observed a uterus, but not an oviduct. These observations might correspond with the report of a study that described a dog with an obviously present uterine structure, despite no oviduct being observed [6].

The two dogs in this report exhibited a testicular tumor in the right abdomen and prostatomegaly. Unilateral cryptorchidism tends to occur on the right side [14] because the right testis, formed more cranially, needs to move a longer distance until it reaches the scrotum than the left one [15], and prostatomegaly is commonly present in PMDS patients [16]. PMDS is known as an inherited condition in the Miniature Schnauzer and Basset Hound breeds and has also been reported in other canine breeds [16, 17]. The coincidence of SCT that has developed from cryptorchidism with PMDS occurs almost solely in the Miniature Schnauzer breed [6, 18]. To the best of our knowledge, this is the first report to describe PMDS with coexisting malignant seminoma or collision tumor in the testes in breeds other than the Miniature Schnauzer.

## Conclusions

It is recommended that Müllerian duct derivatives should be examined in cryptorchidism through ultrasonography and that orchiectomy be performed at an early age for patients that have cryptorchidism to prevent the intra-abdominal testes from becoming tumorous.

## Abbreviations

APTT: Activated Partial Thromboplastin Time
MIF: Müllerian-inhibiting factor
PMDS: Persistent Müllerian duct syndrome
PT: Prothrombin Time
SCT: Sertoli cell tumor
